# Intrinsic and external determinants of antibiotic prescribing: a multi-level path analysis of primary care prescriptions in Hubei, China

**DOI:** 10.1186/s13756-019-0592-5

**Published:** 2019-08-07

**Authors:** Chenxi Liu, Chaojie Liu, Dan Wang, Xinping Zhang

**Affiliations:** 1 School of Medicine and Health Management, Tongji Medical School, Huazhong University of Science and Technology, Wuhan, Hubei China; 2 School of Psychology and Public Health, La Trobe University, Melbourne, Victoria Australia

**Keywords:** Primacy care, Antibiotic prescribing, Path analysis, Knowledge-attitudes-practices, China

## Abstract

**Background:**

Irrational use of antibiotics is a major driver of development of antibiotic resistance, which heavily threatens population health worldwide. Understanding the mechanism of physician’s antibiotic prescribing decisions is increasingly highlighted to promote prudent use of antibiotics. Therefore, the current study aimed to fill the gap, modelling physician’s antibiotic prescribing and identifying the potential intrinsic and external determinants of antibiotic prescribing in primary care.

**Methods:**

A total of 428,475 prescriptions from 499 prescribers in 65 primary care facilities in Hubei of China were audited. Teixeira Antibiotic Prescribing Behavioral Model (TAPBM) was referred as theoretical basis to identify intrinsic and external predictors of antibiotic prescriptions. A questionnaire survey was conducted, covering potential physician’s intrinsic determinants (knowledge, attitudes and individual characteristics) and external factors both in individual level (patient pressure, time pressure and financial incentives) and institutional level (setting and local socio-economic development). A two-level path analysis was performed linking potential determinants of antibiotic use with physician’s actual practices.

**Results:**

About 44.28% of the prescriptions contained antibiotics, with 9.28% containing two or more antibiotics. The multi-level path analysis revealed that knowledge was a significant predictor of attitudes (β = 0.154, *p* < 0.05), but higher knowledge and attitudes failed to translate into antibiotic prescribing practices ((β = − 0.076 – 0.039, *p* > 0.05). Instead, external factors played a more important role and physician’s antibiotic use was significantly associated with patient pressure (β = 0.102, *p* = 0.022), time pressure (β = − 0.164, *p* = 0.002), financial incentives (β = − 0.133– − 0.155, *p* = 0.027) and institutional environments (rural area, β = 0.408, *p* = 0.002; and high socioeconomic setting, β = − 0.641 - -0.578, *p* < 0.001 ). The prescribers who were male (β = − 0.168, *p* = 0.007) or had lower qualification (β = − 0.114, *p* = 0.028) were also more likely to prescribe antibiotics than others.

**Conclusion:**

Antibiotic prescribing practices are complex process and associated with both intrinsic (prescriber) and external (patients and institutional environment) factors. A systematic approach is required to curb over-prescription of antibiotics. Apart from educating prescribers, it is equally important, if not more, to educate patients, break incentives and nurture professional culture within organization to reduce the overuse of antibiotics.

**Electronic supplementary material:**

The online version of this article (10.1186/s13756-019-0592-5) contains supplementary material, which is available to authorized users.

## Backgrounds

Antibiotic resistance (AR), one of the most serious public health issues, threatens population health and socioeconomic development of all nations over the world [1]. It not only jeopardizes our ability to prevent and treat microbial infections, but also put many common medical procedures (e.g. caesarean sections) potentially fatal again, for which antibiotics are commonly used to prevent potential infections. It was projected that AR will contribute to 10 million deaths in 2050 if no effective actions are taken, surpassing cancer and becoming the leading cause of death [2].

Irrational use of antibiotics is a major driver fueling the development of AR [3, 4]. Promoting prudent antibiotic use is considered as a core measure to address this global threat [1]. For patients, antibiotics are commonly prescription-required medicines. Therefore, rational prescribing from physicians plays a fundamental role for prudent use of antibiotics [5]. Unfortunately, inappropriate and over prescriptions of antibiotics are prevalent worldwide. For example, 30% of antibiotic prescriptions in outpatient care in the US were deemed unnecessary [6]. In China, this figure was estimated to be as high as 60% [7].

Many studies have attempted to understand how and why physicians prescribe antibiotics irrationally [8, 9]. Knowledge and attitudes are perhaps the most frequently explored predictors of antibiotic prescribing [10–24]. These studies often suffered from some common methodological limitations. For example, they examined the theoretical knowledge of physicians and their intended use of antibiotics for simulated cases [9, 21]. However, prescribing practices are subject to the influence of a much broader range of factors. Our understanding on actual antibiotic prescribing practices is still limited [8, 9, 25].

Teixeira suggested a comprehensive framework for studying prescribing practices [8]. It considers the influence of both intrinsic factors (e.g. knowledge and attitudes of prescribers) and external factors (e.g. requests from patients, employers and governments) on prescribing practices. The Teixeira Antibiotic Prescribing Behavioral Model (TAPBM) also tries to explain the complicated connections among multiple factors, which may involve precedence or compromise.

Although the TAPBM has been widely endorsed by the research community [8], there is a lack of empirical studies testing the model, possibly due to difficulties to link different sources of data. This study aimed to test the TAPBM through linking the survey data of prescribers to their prescriptions. The findings will contribute to the existing debate on determinants of antibiotic prescribing practices, filling the gap in the literature.

## Methods

### Study setting

This study was conducted in Hubei, a province in central China covering a land of 185.9 thousand km^2^ and 59.02 million populations. Hubei had a gross domestic product (GDP) of $8915 per capita in 2017, ranking at number 11 among all (34) provinces in China. It is classified as a middle-high income region based on the criteria of the World Bank [26].

The study setting was restricted to primary care facilities, which included both urban community health centers (UCHCs) and rural township health centers (RTHCs). In 2017, Hubei had 347 UCHCs and 1137 RTHCs, receiving 23.03 million and 56.00 million outpatient visits, respectively [26]. Over-prescription of antibiotics was prevalent in these facilities: 65% of prescriptions contained antibiotics and 20% involved two or more antibiotics [27].

### Theoretical framework

This study adopted the TAPBM as an overarching framework. The TAPBM extended the widely-accepted Knowledge-Attitudes-Practices (KAP) theory and included considerations of external determinants of antibiotic prescribing practices [8].

*Intrinsic determinants:* knowledge and attitudes are the core of intrinsic determinants of antibiotic prescribing practices. Knowledge can influence prescribing behaviors directly or indirectly through influencing attitudes [8]. The knowledge and attitudes of prescribers can be shaped by many individual characteristics [8, 9], such as gender, qualifications, clinical expertise, continuing education, and years of practices.

*External determinants:* when physicians prescribe medicines, they are likely to take considerations of many external factors apart from relevant clinical standards and guidelines. Pressures from patients, peers, employers, governments, and the public may sway antibiotic prescribing decisions. These factors can exert impacts on prescribing practices directly or indirectly through influencing attitudes [8].

Previous studies showed that patient requests for antibiotics increased antibiotic prescribing dramatically, especially when the physicians felt that they did not have enough time to explain details to their patients [28, 29]. It has been well documented that financial incentives can also drive antibiotic prescribing in primary care in China. Such perverse incentives are particularly strong for the physicians with a lower household income [30].

Formal and informal institutional and system arrangements also play an important role in shaping prescribing decisions. Several studies in the European countries showed that antibiotic prescribing practices are associated with the culture of an organization (shared values and behaviors among employees) [31, 32] and the culture of a nation (e.g. tolerance of uncertainty and status distinction) [33]. Empirical evidence in China showed that over-prescription of antibiotics is more prevalent in primary care facilities in rural [7] and socioeconomically disadvantaged regions [34, 35] compared with those in urban and better-off regions.

**Fig. 1:**
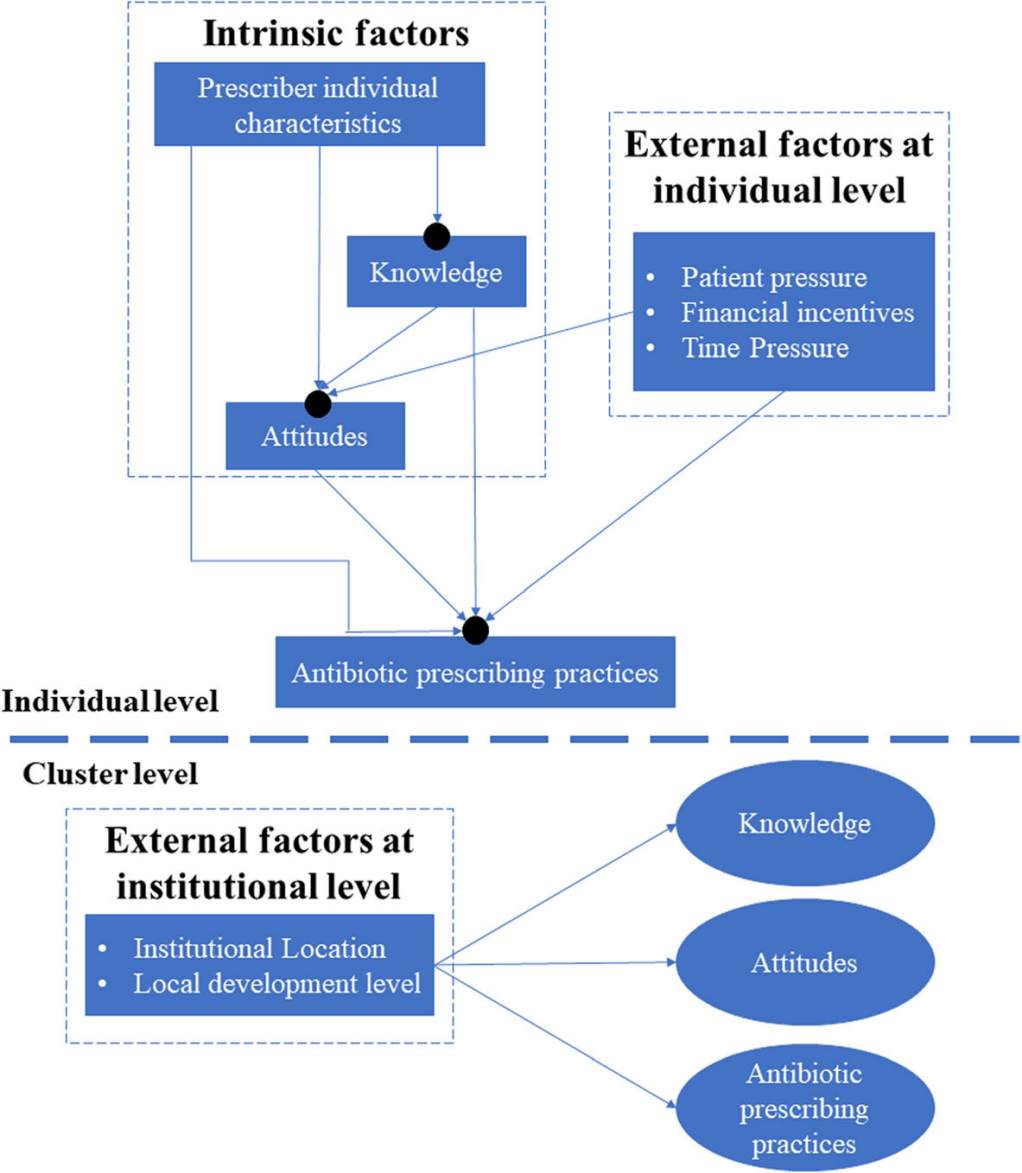
Antibiotic prescribing behavioral model adapted from the TAPBM. Institutional variations in individual knowledge, attitudes and prescribing practices were treated as a random effect and depicted using black dots. Their random intercepts were predicted by two institutional indicators

In this study, the TAPBM framework was adapted considering the availability of relevant data and measurements. We adopted a two-level modelling approach, covering the effects of individual (physician) factors and institutional (cluster-level) factors. The individual-level measurements included both intrinsic (e.g. characteristics of prescribers) and external (e.g. perceived pressures) indicators. The institutional-level measurements were represented by two proxy indicators: urban/rural location and socioeconomic status of the servicing community (Fig. 1). We chose a two-level model for two reasons. First, a cluster sampling strategy was adopted to select study participants. Second, cluster effects were significant as measured by the intra-cluster correlation coefficient (ICC): 0.004 for knowledge, 0.094 for attitudes, and 0.395–0.408 for antibiotic prescribing practices. Institutional variations in individual knowledge, attitudes and prescribing practices were deemed random (depicted using a black dot in Fig. 1), and their random intercepts were predicted by the two institutional indicators.

### Measurements

#### Outcome measures

Two indicators were calculated to measure antibiotic prescribing practices in line with the World Health Organization (WHO) recommendations [36]:

- Percentage of prescriptions containing antibiotics = Number of prescriptions containing antibiotics / Total prescriptions × 100%;
- Percentage of prescriptions with combined use of antibiotics = Number of prescriptions containing two or more antibiotics / Total prescriptions × 100%;

#### Predictors of outcome measures

##### Individual-level intrinsic factors

*Knowledge*: Antibiotic prescribing knowledge was measured with 11 items that were commonly used in previous studies [13, 22, 37], asking the physician respondents to make a prescribing decision under various scenarios, such as for patients with non-febrile diarrhea, upper respiratory tract infections, methicillin resistant staphylococcus, and bacterial pneumonia. Each scenario was provided with 4 or 5 prescribing options, with only one correct answer. An “unknown” option was also listed to discourage guessing. A summed score of knowledge about antibiotic prescribing was calculated, ranging from 0 to 11. A higher score indicates better knowledge.

*Attitudes:* Attitudes of the physician respondents were measured using the 11-item Knowledge and Attitudes regarding Antibiotics and Resistance (KAAR-11) scale, which has been fully validated in previous studies [38]. The KAAR-11 asked respondents to rate their opinions on a four-point Likert scale (ranging from 0 “totally agree” to 4 “totally disagree”) about the excuses for irrational prescriptions of antibiotics, such as satisfying patient requests, avoiding patient complaints, ignoring or blaming others for AR. A summed score of KAAR-11 was calculated, ranging from 0 to 44. A higher score indicates a more favorable attitude to rational prescribing of antibiotics [38].

*Individual characteristics of prescribers*: These included the demographic characteristics (gender and education) and the professional experiences (years of clinical practices, professional title, and training on antibiotic prescriptions) of the participants. All of the physician respondents were general practitioners. Therefore, clinical expertise was excluded in the analysis.

##### Individual-level external factors

*Perceived patient pressure*: Respondents were asked “how many percent of patients you see expect antibiotic prescriptions” and “how often the patient expectation influences your decision of antibiotic prescribing”. The latter was rated on a five-point Likert scale ranging from “1 = always” to “0 = never”. Scores of the two questions were multiplied as an indication of patient pressure (ranging from 0 to 100).

*Time pressure*: Respondents were asked to estimate the average duration (minutes) of consultations for each patient visit, with a lower duration indicating higher time pressure.

*Household income*: Respondents were asked to estimate their annual household income (Chinese Yuan ¥) over the previous year. The household income was classified into four categories: < 40,000; 40,000-79,999; 80,000-119,999; ≥120,000.

##### Institutional-level measures

The characteristics of the participating primary care institutions were measured with two indicators: rural/urban location and socioeconomic status of the servicing community. All of the participating primary care facilities were public institutions governed by the same policy and regulatory framework. The socioeconomic status was classified based on GDP per capita of their local districts: high (top four districts) vs low.

#### Pilot survey

The questionnaire was pilot tested in 23 physicians from 3 primary care facilities. They were asked to explain why they chose or did not choose certain answers. The feedback was used to modify the questionnaire. Of the 30 tested questionnaire items, 9 were rephrased and no items were deleted or added (the final instrument could be found in the Additional file 1: Table S1).

### Sampling and data collection

A stratified cluster sampling strategy was adopted to select study participants. A total of 65 primary care facilities were randomly selected from 3 urban and 6 rural districts corresponding to the distribution of primary care facilities in Hubei. The participating primary care institutions represented 5.5% (19/347) of UCHCs and 4.1% (46/1137) of RTHCs, respectively. Details about the sampling approach have been published elsewhere [39].

The physicians who prescribed antibiotics independently in the participating primary care facilities were eligible for this study. To minimize bias, only those who prescribed ≥100 prescriptions three months prior to the commencement of the questionnaire survey of prescribers were invited to participate in this study.

#### Extraction of prescription data

Prescription data from January 1st to March 31st, 2018 were extracted either from the participating primary care institutions or from the medical administration system of the local governments. These included the name of the institution, the ID number of the prescriber, patient ID, date of the prescription, and the type, volume and price of the medicines prescribed.

#### Questionnaire survey

A questionnaire survey of prescribers in the participating primary care institutions was conducted from April 23rd to June 6th, 2018. A pair of trained investigators visited each primary care facility and invited all of the physicians working at the time to complete the questionnaire. An informed written consent was obtained before the respondents self-completed the questionnaire. The survey took about 15 min. Completed questionnaires were returned to the investigators immediately. The investigators examined the completeness of the returned questionnaires. Missing items, if existed, were re-filled through a supplementary interview. A token gift ($1.65) was given to the participants.

In total, 712 physicians agreed to participate in the survey, representing 93.44% of all physicians in the participating institutions. About 664 complete questionnaires were returned, of which 499 met the inclusion criteria and passed logic check. This resulted in an effective response rate of 70.08%.

#### Data mapping

The prescription outcome indicators were calculated from the extracted prescription database and then mapped into the questionnaire survey data in line with the ID number of the prescribers. The two institutional variables were obtained from the classification system of the statistics bureau of Hubei government and mapped into the questionnaire data in line with the name of the institutions.

### Data analysis

We compared the differences of respondents between the urban and rural settings, using Chi-square (or Fisher’s exact) tests for binary variables, Wilcoxon rank-sum tests for continuous variable without a normal distribution, and Student *t* tests for continuous variables with a normal distribution.

A two-level path analysis was performed to test the TAPBM using a mixed effect model. Institutional variations in the individual knowledge, attitudes and prescribing practices were treated as a random effect, with the random intercepts being further predicted by the institutional (cluster-level) variables. The analysis adopted the maximum likelihood with robust standard error (MLR) estimation, which can be applied for variables without a normal distribution. The fitness of data into the model was assessed using three criteria [40, 41]: RMSEA< 0.08, CFI > 0.95, and SRMR< 0.08.

The statistical analyses were performed using STATA (version 12.0) and Mplus (version 6.0). A *p* value < 0.05 was considered statistically significant.

## Results

### Characteristics of survey respondents

About 21.64% of respondents worked in the UCHCs. Over half of the UCHC respondents were women compared with only 23.53% in the RTHCs (*p* < 0.001). Compared with the UCHC respondents, those from the RTHCs were younger, had lower qualifications, lower professional titles, shorter experiences in clinical practices, and lower household income (Table 1). Over three quarters (78.76%) of the RTCH respondents attended training programs on antibiotic prescribing in 2017, compared with 62.96% of respondents from the UCHCs (*p* = 0.001).

**Table 1:** Characteristics of study participants

Characteristics	Overall	Urban Community Health Center	Rural Township Health Center	p*
Number of respondents	499	108	391	–
Gender, n (%)				< 0.001
Men	352 (70.54)	53 (49.07)	299 (76.47)	
Women	147 (29.46)	55 (50.93)	92 (23.53)	
Age, Years (Mean ± SD)	43.38 ± 9.59	48.37 ± 10.26	42.00 ± 8.93	< 0.001
Years of clinical practices	16.28 ± 10.10	18.69 ± 11.40	15.61 ± 9.62	0.015
Qualification				< 0.001
No degree	42 (8.42)	8 (7.41)	34 (8.70)	
Associate degree	266 (53.31)	38 (35.19)	228 (58.31)	
University degree	191 (38.28)	62 (57.41)	129 (32.99)	
Professional title				< 0.001
Junior	257 (51.50)	28 (25.93)	229 (58.57)	
Middle	191 (38.28)	53 (49.07)	138 (35.29)	
Senior	51 (10.22)	27 (25.00)	24 (6.14)	
Annual household income (¥^+^)				< 0.001
< 40,000	143 (28.66)	17 (15.74)	126 (32.23)	
40,000-79,999	253 (50.70)	47 (43.52)	206 (52.69)	
80,000–119,999	77 (15.43)	29 (26.85)	48 (12.28)	
≥ 120,000	26 (5.21)	15 (13.89)	11 (2.81)	
Training on antibiotic prescribing in 2017				0.001
Attended	374 (74.95)	68 (62.96)	306 (78.26)	
Not attended/Not aware	125 (25.05)	40 (37.04)	85 (21.74)	

^*^Wilcoxon-Mann-Whitney tests (continuous and ordinal variables) or Chi-square tests (binary variables); ^+ ^¥ represented the Chinese unit of currency, Renminbi (RMB)

### Antibiotic prescribing practices

The study participants prescribed 428,475 prescriptions over the three-month period: 44.28% (189,719) contained antibiotics and 9.28% (39,778) contained two or more antibiotics. Those from the RTHCs were more likely to prescribe antibiotics than their UCHC counterparts. But the differences were only statistically significant in the combined use of antibiotics (*p* < 0.001) (Table 2).

**Table 2:** Antibiotic prescriptions and associated factors in primary care

	Overall	UCHCs	RTHs	*p* *
*Antibiotic prescribing practices of individual physicians (Mean ± SD* ***)***				
Percentage of prescriptions containing antibiotics (%)	41.45 ± 20.13	39.55 ± 23.35	41.97 ± 19.15	0.066
Percentage of prescriptions containing two or more antibiotics (%)	10.23 ± 10.53	7.00 ± 9.82	11.12 ± 10.56	**< 0.001**
*Number (%) of correct answers to antibiotic prescribing knowledge*				
K1: Non-febrile diarrhea	475 (95.19)	102 (94.44)	373 (95.40)	0.682
K2: Upper respiratory tract infections	25 (5.01)	3 (2.78)	22 (5.63)	0.320
K3: Renal failure	56 (11.22)	8 (7.41)	48 (12.28)	0.156
K4: Pregnant patients	482 (96.59)	104 (96.30)	378 (96.68)	0.770
K5: Anaerobes	485 (97.19)	108 (100.00)	377 (96.42)	**0.048**
K6: Methicillin resistant staphylococcus	145 (29.06)	35 (32.41)	110 (28.13)	0.386
K7: Crossing the blood-brain barrier	206 (41.28)	50 (46.30)	156 (39.90)	0.232
K8: Bacterial pneumonia	232 (46.49)	56 (51.85)	176 (45.01)	0.207
K9: Reducing complications of upper respiratory tract infections	263 (52.71)	56 (51.85)	207 (52.94)	0.841
K10: Administration of Aminoglycosides	305 (61.12)	69 (63.89)	236 (60.36)	0.505
K11: Standards of antibiotic use in primary cares	375 (75.15)	93 (86.11)	282 (72.12)	**0.003**
Summed knowledge score (Mean ± SD)	6.11 ± 1.46	6.33 ± 1.42	6.04 ± 1.46	0.073
*Score of attitudes toward rational antibiotic prescribing (Mean ± SD)*				
A1: Antibiotic resistance is a major public health problem in my setting	3.02 ± 0.90	2.79 ± 1.01	3.09 ± 0.86	**0.006**
A2: It is useful to wait for a microbiology result when treating infections	3.34 ± 0.60	3.34 ± 0.66	3.34 ± 0.59	0.710
A3: One antibiotic prescription does not influence the development of AR	3.04 ± 0.82	2.98 ± 0.96	3.06 ± 0.78	0.781
A4: New antibiotics will be created to solve AR problems	1.54 ± 0.94	1.74 ± 1.07	1.48 ± 0.89	**0.017**
A5: The use of antibiotics in animals is a major cause of AR	2.60 ± 0.91	2.71 ± 0.81	2.57 ± 0.94	0.219
A6: Broad-spectrum antibiotics are preferred for infections in doubt	1.99 ± 1.02	2.22 ± 1.02	1.92 ± 1.01	**0.007**
A7: Antibiotics are often prescribed for patients untrackable	2.89 ± 0.96	3.05 ± 0.95	2.85 ± 0.96	**0.035**
A8: It is best to prescribe antibiotics if bacterial infections are uncertain	2.87 ± 0.83	3.01 ± 0.80	2.83 ± 0.84	**0.042**
A9: Antibiotics are often prescribed due to patient demands	2.86 ± 0.91	2.77 ± 0.88	2.88 ± 0.92	0.161
A10: Patients will get antibiotics from a pharmacy even without my prescriptions	1.19 ± 0.94	1.19 ± 0.96	1.20 ± 0.93	0.938
A11: Amoxicillin is effective for most respiratory infections in primary care	2.22 ± 1.01	2.25 ± 1.05	2.21 ± 1.00	0.737
Summed attitudes score	27.56 ± 3.46	28.05 ± 3.52	27.42 ± 3.43	0.097
*Perceived patient pressure (Mean ± SD)*				
Percentage of patients expecting antibiotics	54.91 ± 22.59	50.23 ± 21.48	56.20 ± 22.75	**0.001**
Degree of impacts of patient expectation on antibiotic prescribing	43.34 ± 26.41	40.74 ± 25.48	44.05 ± 26.64	0.204
Total score of perceived patient pressure for antibiotics	25.63 ± 21.41	22.22 ± 21.40	26.57 ± 21.47	**0.026**
*Time pressure (Mean ± SD)*				
Length of consultant per visit (Minutes)	10.58 ± 6.47	12.00 ± 6.91	10.18 ± 6.30	**0.007**

*Chi-square (fisher exact) tests for binary variables, Wilcoxon rank-sum tests for continuous variable without normal distribution and t tests for continuous variable with normal distribution; Boldface figures indicate the significant differences between physicians in UCHCs and those in RTHs

The respondents had a mean knowledge score of 6.11 (SD = 1.46) about rational antibiotic prescriptions. The percentage of respondents giving a correct prescribing answer was very low: 5.01% for upper respiratory tract infections, 11.22% for renal failure, 29.06% for methicillin resistant staphylococcus, 46.49% for bacterial pneumonia, and 41.28% for antibiotic treatments crossing the blood-brain barrier. No overall knowledge difference appeared between the UCHC and RTHC respondents (*p* = 0.073), although those from the UCHCs were more likely to give a correct answer to antibiotic prescriptions for anaerobes (*p* = 0.048) and to the standards of antibiotic use in primary care (*p* = 0.003).

The respondents had a mean KAAR-11 score of 27.56 (SD = 3.46). Compared with the UCHC respondents, those from the RTHCs were more likely to recognize AR as a public health problem (*p* = 0.006), but blame others for the development of AR (*p* = 0.017), and prescribe antibiotics for defensive purpose (*p* < 0.05).

The study participants perceived high levels of patient pressure on antibiotic prescribing. On average, over half (54.91%) of the patients were reported to have an expectation on antibiotic prescriptions. This resulted in a mean patient pressure score of 25.63% (SD = 21.41%) weighted by perceived influence of patient requests. The RTHC physicians perceived higher patient expectations (*p* = 0.001) and greater impacts of patient pressure (*p* = 0.026) compared with their UCHC counterparts.

On average, the physician respondents spent 10.58 min (SD = 6.47) for each patient consultation. The length of patient consultation was longer in the UCHCs than in the RTHCs (*p* = 0.007), indicating a lower time pressure.

### Determinants of antibiotic prescribing behaviors

**Fig. 2:**
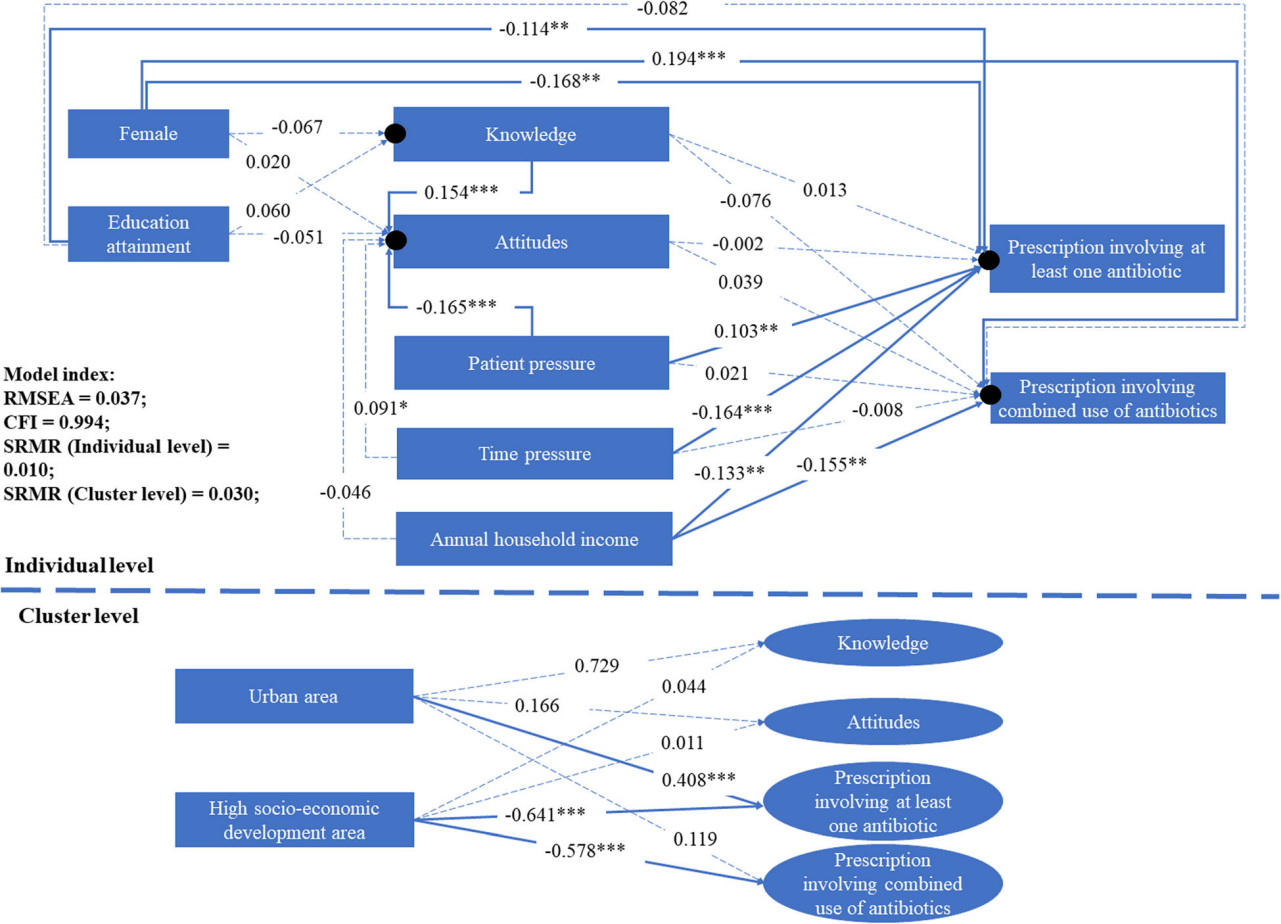
Determinants of physician’s antibiotic prescribing based on TAPBM using two-level path analysis. The black dots indicate the random intercepts of knowledge, attitudes and prescribing practices that are predicted by the cluster-level variables. *: *p* < 0.10; **: *p* < 0.05; ***: *p* < 0.01

A good fitness of the data into the TAPBM model (Fig. 2) was evident: RMSEA = 0.037 (< 0.08), CFI = 0.994 (> 0.95), SRMR< 0.08 at both individual and cluster levels.

Intrinsic factors: individual knowledge and attitudes did not predict antibiotic prescribing practices (β: − 0.076–0.039, *p* > 0.05), although better knowledge was associated with positive attitudes toward rational use of antibiotics (β = 0.154, *p* = 0.001). Female physicians were less likely to prescribe antibiotics (β = − 0.168, *p* = 0.007) but more likely to use two or more antibiotics (β = 0.194, *p* < 0.001) compared with their male counterparts. Higher qualifications were associated with less prescriptions of antibiotics (β = − 0.114, *p* = 0.028). Professional title, experience of clinical practices, and continuing education were not significant predictors (*p* > 0.05) on knowledge, attitudes and practices in antibiotic prescribing (omitted in Fig. 2).

External factors: perceived higher patient pressure was associated with negative attitudes towards rational use of antibiotics (β = − 0.165, *p* < 0.001) and higher use of antibiotics (β = 0.102, *p* = 0.022). Higher time pressure was associated with higher use of antibiotics (β = − 0.164, *p* = 0.002), although it failed to predict attitudes toward rational use of antibiotic (β = − 0.091, *p* > 0.05). Higher household income was associated with lower use of antibiotics (β = − 0.133, *p* = 0.027) and lower combined use of antibiotics (β = − 0.155, *p* = 0.012).

At the cluster-level, rural location was a significant predictor of higher use of antibiotics (β = 0.408, *p* = 0.002). The facilities servicing communities with a higher socioeconomic status were less likely to prescribe antibiotics (β = − 0.641, *p* < 0.001) and less likely to use combined antibiotics (β = − 0.578, *p* < 0.001). The institutional variables did not predict the knowledge and attitudes of physicians (*p* > 0.05).

## Discussions

### Main findings

This study confirmed that antibiotic prescribing practices involve complex mechanisms and the TAPBM can be used for exploring determinants of antibiotic prescriptions, including both intrinsic and external factors at the individual and institutional levels.

High levels of antibiotic prescriptions are evident in primary care in Hubei: 44.28% involved at least one antibiotic. Although this represents a decline of antibiotic prescriptions in general [27], it is still much higher than what has been recommended by the Chinese government (20%) and the World Health Organization (30%) [42]. In this study, the physicians demonstrated poor knowledge about antibiotic prescribing. Although poor knowledge does not predict antibiotic prescribing directly, it may leave greater opportunities for external factors to sway prescribing decisions. We found that time pressure and patient requests are associated with higher levels of antibiotic prescriptions. Antibiotic prescribing practices are also associated with individual characteristics (gender, education and income) of physicians and institutional environments (location and socioeconomic status).

### Strengths and weaknesses of the study

This study tested both intrinsic and external determinants of antibiotic prescribing through a two-level path analysis based on the TAPBM. Such an approach allowed us to examine antibiotic prescribing practices in a comprehensive and systematic way, tapping into factors associated with prescribers, patients, and institutional environments [8]. We were able to link prescribing data with survey data on prescribers, which has commonly been absent in previous studies [10–24]. We also adopted well-validated instruments for measuring knowledge and attitudes of physicians, addressing problems embedded in some previous studies [43].

There are several limitations in this study. Firstly, this study was conducted in Hubei, a middle-high income region. Generalization of the findings of this study to other regions should be cautious. Secondly, in this study, prescribing indicators were not risk-adjusted due to unavailability of data regarding patient conditions. Thirdly, there is a consensus that the pharmaceutical industry may play an important role in driving prescribing decisions of physicians. The Chinese government has attempted to delink financial incentives associated with prescribing practices. However, measuring such links is difficult, if not impossible. We could only use household income as a proxy indicator in this study. Finally, there is large standard deviation of some parameters in current study, indicating a huge difference in physician’s knowledge, attitudes and practices. The situation may imply that several latent classes of physicians, and among which a specific group of physicians with poorer knowledge and attitudes may play an important role contributing to the high irrational prescribing of antibiotics. However, the analysis is out of reach of the current study and further studies are warranted.

### Comparison to other studies

#### Effects of intrinsic factors

Knowledge and attitudes may play different roles in prescribing practices under different system contexts. This study found no direct links between “knowledge and attitudes” and antibiotic prescribing practices, similar to the findings of a study of general practitioners (GPs) in Scotland [16]. But in Spain, appropriate use of antibiotics in clinical practices was found to be closed associated with the specific knowledge and attitudes of primary care physicians [21].

Clearly, interventions targeting prescribers alone on their knowledge and attitudes are not enough to curb over-prescriptions of antibiotics. In another study using the theory of planned behavior, we also found that antibiotic prescribing is not under the volitional control of physicians [39].

Individual characteristics of prescribers can shape antibiotic prescribing practices. Several studies revealed that female physicians are less likely to prescribe antibiotics [44, 45], consistent with the findings of this study. But female prescribers are more likely to combine antibiotics when they prescribe antibiotics. Roter and colleagues concluded in a systematic review that such a prescribing style is likely to be associated with the more patient-centered communication style of female physicians [46]. Female prescribers found it easier to implement the “wait-and-see” policy on antibiotic prescribing but harder to refuse patient requests compared with their male counterparts [45].

Higher medical qualifications were found to be associated with less antibiotic prescriptions in this study after adjustments of knowledge levels. This may be attributable to the professional culture. Formal and systematic medical training may boost the confidence of practitioners [47] and encourage physicians to consider a greater variety of interventional strategies [48].

#### Effects of external factors

It appears that external factors play a more important role in predicting antibiotic prescribing. Similar to findings of other studies [25, 49–52], this study revealed that patient pressure is a major driver of antibiotic prescriptions. In China, defensive clinical practices are common [53]. The poor health literacy of consumers and a lack of trust in medical service providers is often blamed for fueling over-prescriptions of antibiotics.

There is no direct evidence in this study to prove that antibiotic prescribing practices are associated with financial incentives. However, low household income of physicians was found to be associated with high antibiotic prescriptions. Such a link was also documented in some previous studies [54]. Despite a significant increase in governmental investment on primary care in China, two-thirds of the budget of primary care facilities still needs to be recovered by service revenues [54]. This may motivate primary care physicians to seek extra income through over-provision of services.

The institutional variations in antibiotic prescribing practices are concerning. Rural health facilities and those servicing communities with a low socioeconomic status are relatively poorly resourced. But they are more likely to prescribe antibiotics than their better-off counterparts. China is not alone. Similar phenomena were also observed in Italy [34], Switzerland [55] and Germany [56]. This has significant implications on the effective use of the already scant health resources in the worse-off communities [57].

### Policy implications

Policy interventions targeting external factors associated with antibiotic prescribing are essential. Although medical education by itself is important [58], it can only be adopted as a long term strategy. The current focus and investment on continuing education [59] and development of clinical guidelines [60] will have limited effects if external determinants of antibiotic prescribing are not properly addressed [7, 35, 61, 62].

To address the external determinants revealed in this study, several interventional strategies should be highlighted according to the empirical evidence published in the literature:

(1) Better communication skills and “wait-and-see” approach may help physicians to deal with patient pressure for antibiotics [63–65]. A study in Guangxi of China confirmed the value of communication training programs in reducing antibiotic prescriptions in pediatric care [66].

(2) Patients can be educated through social marketing campaigns to improve their understanding on the use of antibiotics and subsequently reduce requests for antibiotics [67]. However, it is challenging to change the public stereotype of antibiotics as panacea. Some pilot trials in patient education using leaflets [66] or public reporting [68] have demonstrated limited efforts in China.

(3) There is a need to improve governance and management competencies in primary care. The funding system should encourage prescribers to make decisions based on the clinical conditions of their patients, not financial gains [69, 70]. A professional culture needs to be nurtured. Empirical evidence shows that antibiotic prescribing decisions are often influenced by peers. Antibiotic prescribing can be significantly reduced when a prescriber perceives her/himself as an outlier compared with colleagues [71].

## Conclusion

Antibiotic prescribing is a complex process shaped by both intrinsic (knowledge, attitudes and individual characteristics) and external (patients, institutions and healthcare systems) factors. A systems approach is required to curb over-prescription of antibiotics in China. Apart from educating prescribers, it is equally important, if not more, to educate patients to ease the pressure from patient requests. Management also plays an important role in changing prescribing behaviors.

The link between high use of antibiotics and low socioeconomic status is concerning. Further studies are warranted to explore the underlying reasons of higher antibiotic use in rural and low socioeconomic settings in China.

## Additional file

Additional file 1: **Table S1.** Survey instrument. (DOCX 33 kb)

## Data Availability

The data that support the findings of this study are available from surveyed local institutions and governments but restrictions apply to the availability of these data, which were used under license for the current study, and so are not publicly available. Data are however available from the authors upon reasonable request and with permission of surveyed local institutions and governments.

## Abbreviations

AR: Antibiotic resistance
CFI: comparative fit index
GDP: Gross domestic product
ICC: Intra-cluster correlation coefficient
KAAR-11: 11-item Knowledge and Attitudes regarding Antibiotics and Resistance
KAP: Knowledge-attitudes-practices
MLR: Maximum likelihood with robust standard error
RMSEA: Root mean square error of approximation
RTHCs: Rural township health centers
SD: Standard deviation;
SEM: Structural equation modelling;
SRMR: Standardized root mean squared residual
TAPBM: Teixeira Antibiotic Prescribing Behavioral Model
UCHCs: Urban community health centers
WHO: World Health Organization
