# Supplementary material for: Intrinsic and external determinants of antibiotic prescribing: a multi-level path analysis of primary care prescriptions in Hubei, China
Source: Antimicrob Resist Infect Control. 2019 Aug 7;8:132. doi: 10.1186/s13756-019-0592-5 (PMC6686458; doi:10.1186/s13756-019-0592-5)
Supplement: Supplementary file 1 — Table S1. Survey instrument. (DOCX 33 kb) [file 13756_2019_592_MOESM1_ESM.docx]

**Survey of influencing factors of antibiotic prescribing in primary cares**

**Dear physicians：**

Greeting!

We are conducting a survey regarding to physicians’ knowledge, attitudes and behaviors of antibiotic prescribing. We invited you to complete this 15-minute questionnaire and your responses are of great values to help us understand physicians’ antibiotic prescribing behaviors. Your personal information would be confidential, and the responses were only used for academic research only. Thanks very much for your participation.

School of Medicine and Health Management

Tongji Medical School, Wuhan

**Part 1: Knowledge**

Note: this part of the questions **only has one correct answer**

**1. A 4-year-old girl has diarrhea for 4 days (3 stools/day). She has no fever at examination nor during the last few days. Which treatment do you propose?**

a) amoxicillin p.o.;  b) TMP/SMX ** p.o.;  c) amoxicillin-clavulanic acid p.o.;

d) no antibiotic treatment, only oral rehydration; e) I don’t know

**2. A 6-year-old child has a fever of 38°C, purulent rhinitis and angina for two days. At inspection, the throat is reddish. Which treatment do you recommend?**

a) TMP/SMX ** p.o.  b) amoxicillin p.o.  c) amoxicillin-clavulanic acid p.o.

d) no antibiotic e) I don’t know

**3. During your ward round, you see two patients with severe renal failure. Patient A is a 68-year-old man suffering from serious cellulitis at the leg, he is treated with clindamycin. Patient B is a 64-year-olddiabetic woman which is blindly (empirically) treated for septicemia with ceftriaxone. Dosage reduction is needed for:**

a) Patient A  b) Patient B  c) both patients

d) in neither patient A nor patient B e) I don’t know

**4. Which one of the following antibiotics is safe during pregnancy?**

a) amoxicillin  b) ciprofloxacin  c) gentamicin d) I don’t know

**5. Which one of the following antibiotics has the best activity against anaerobes?**

a) ciprofloxacin   b) metronidazole c) cotrimoxazole d) I don’t know

**6. Methicillin resistant - Staphylococcus aureus is susceptible to:**

a) amoxicillin-clavulanic acid  b) cefotaxime  c) ceftriaxone

d) none of those antibiotics e) I don’t know

**7. Which one of the following antibiotic most effectively crosses the blood-brain barrier?**

a) clindamycin   b) ceftriaxone  c)vancomycin d) I don’t know

**8. Aminoglycosides such as gentamicin are very active if they are administered as follows :**

a) orally three times daily  b) parenteral once daily c) parenteral three times daily d) I don’t know

**9. In case of having respiratory infectious symptoms, when does the child need antibiotic?**

a) Cough, stuffy nose or runny nose without fever b) Cough, stuffy nose or runny nose with fever

c) Including one of the following symptoms: fast breathing, chest in-drawing or stridor d) both b) and c)

e) I don’t know

**10. Which of the following statements is correct?**

a) Antibiotics are helpful in treating URTIs b) Antibiotics reduce the duration of URTIs

c) Antibiotics can reduce the occurrence of complications of URTIs d) None of the above

e) I don’t know

**11. According to international recommendations, the average number of prescriptions containing antibiotics per 100 should be _____ in a primary care facility (one prescription per patient)?**

a) below 30 b) 30 – 50 c) 50 – 70 d) above 70 e) I don’t know

**Part 2: Attitudes**

Note: this part of the questions **does not have the correct answer**, please fill in them according to your personal situation / attitude.

| To what extent you agree with the following statements | Very agree | Agree | Neutral | Disagree | Very Disagree |
| --- | --- | --- | --- | --- | --- |
| 1. Antibiotic resistance is a major public health problem in our setting | □ | □ | □ | □ | □ |
| 2. In primary care it is useful to wait for a microbiology result when treating infectious diseases | □ | □ | □ | □ | □ |
| 3. The prescription of an antibiotic to a patient does not influence the development of resistance | □ | □ | □ | □ | □ |
| 4. New antibiotics will be developed to solve the problem of resistance | □ | □ | □ | □ | □ |
| 5. The use of antibiotics in animals is a major cause of the occurrence of new resistance | □ | □ | □ | □ | □ |
| 6. When in doubt, it is better to ensure that a patient is cured of an infection by using a broad-spectrum antibiotic | □ | □ | □ | □ | □ |
| 7. Antibiotics are often prescribed because it is impossible to track the patient accurately | □ | □ | □ | □ | □ |
| 8. When in doubt as to whether a patient has a bacterial disease, it is best to prescribe an antibiotic | □ | □ | □ | □ | □ |
| 9. Antibiotics are often prescribed due to patients’ demands | □ | □ | □ | □ | □ |
| 10. If patients believe that they need an antibiotic and the doctor does not prescribe it, they will get it at the pharmacy without a prescription | □ | □ | □ | □ | □ |
| 11. Amoxicillin is useful for resolving most respiratory infections in primary care | □ | □ | □ | □ | □ |

**Part 3: External factors**

Note: this part of the questions **does not have the correct answer**, please fill in them according to your personal situation.

| Items | Answers |
| --- | --- |
| 1. Approximately, what is the number of patients seen per day? | _______ patients |
| 1. Approximately, how much time do you need to attend one patient? | _______ minutes |
| 1. How many percent of patients that you perceived expect antibiotic prescriptions? | □ All (100%) □ Most (75%) □ Half (50%) □ Part (25%) □ None (0%) |
| 1. How often patient expectation of antibiotic prescriptions influence you prescribing decision | □ Always influential (100%) □ Mostly influential (75%) □ Partly influential (50%) □ A little influential (25%) □ Never influential (0%) |

**Part 4: Personal characteristics**

| 1. Age | years |
| --- | --- |
| 2. Gender | □Male □Female |
| 3. Working place | □ Community healthcare centers □Township hospitals |
| 4. Job title | □ Doctor □ Attending doctor □ Associate chief doctor □ Chief doctor |
| 5. Education | □Secondary school and below □High school □College □Undergraduate □Postgraduate and above |
| 6. Family annual income (￥) | □<20,000 □20,000 – 40,000 □40,000 – 60,000 □60,000 – 80,000 □80,000 – 100,000 □ 100,000 – 120,000 □120,000 – 140,000 □140,000 – 160,000 □160,000 – 180,000 □180,000 – 200,000 □> 200,000 |
| 7. Years of practices | years |
| 8. Whether received training of antibiotics in the last year | □Yes □No/I don’t know |
